# Molecular Analysis of Twist1 and FGF Receptors in a Rabbit Model of Craniosynostosis: Likely Exclusion as the Loci of Origin

**DOI:** 10.1155/2013/305971

**Published:** 2013-05-08

**Authors:** Phillip H. Gallo, James J. Cray, Emily L. Durham, Mark P. Mooney, Gregory M. Cooper, Sandeep Kathju

**Affiliations:** ^1^Department of Plastic Surgery, University of Pittsburgh, Pittsburgh, PA 15261, USA; ^2^Department of Oral Biology, Georgia Health Sciences University, Augusta, GA 30912, USA; ^3^Departments of Anthropology and Orthodontics, University of Pittsburgh, Pittsburgh, PA 15261, USA; ^4^Department of Oral Biology, University of Pittsburgh, Pittsburgh, PA 15261, USA; ^5^Department of Bioengineering, University of Pittsburgh, Pittsburgh, PA 15261, USA

## Abstract

Craniosynostosis is the premature fusion of the cranial vault sutures. We have previously described a colony of rabbits with a heritable pattern of nonsyndromic, coronal suture synostosis; however, the underlying genetic defect remains unknown. We now report a molecular analysis to determine if four genes implicated in human craniosynostosis, TWIST1 and fibroblast growth factor receptors 1–3 (FGFR1–3), could be the loci of the causative mutation in this unique rabbit model. Single nucleotide polymorphisms (SNPs) were identified within the Twist1, FGFR1, and FGFR2 genes, and the allelic patterns of these silent mutations were examined in 22 craniosynostotic rabbits. SNP analysis of the Twist1, FGFR1, and FGFR2 genes indicated that none were the locus of origin of the craniosynostotic phenotype. In addition, no structural mutations were identified by direct sequence analysis of Twist1 and FGFR3 cDNAs. These data indicate that the causative locus for heritable craniosynostosis in this rabbit model is not within the Twist1, FGFR1, and FGFR2 genes. Although a locus in intronic or flanking sequences of FGFR3 remains possible, no direct structural mutation was identified for FGFR3.

## 1. Introduction

Craniosynostosis (CS) is the premature fusion of one or more of the fibrous joints of the calvaria (cranial sutures). If this synostosis happens early enough in human development, it can lead to alterations in skull shape, reduced cranial growth, increased intracranial pressure, impaired blood flow, impaired vision and hearing, as well as mental retardation [1–7]. In most cases, surgical intervention is necessary to improve the patient's prognosis [8–11]. There are extensive signaling networks present within the cranial sutures that allow for the coordinated growth of the skull [12]. One such network involves the fibroblast growth factor receptors (FGFRs). FGFRs belong to a family of tyrosine kinase receptors that exhibit a common organization, including two or three extracellular immunoglobulin (Ig) like binding domains, a transmembrane domain, and two intracellular tyrosine kinase subdomains [13]. The binding of FGF to FGFR in association with heparin sulphate proteoglycan (HSPG) induces receptor dimerization at the cell surface. This dimerization in turn leads to autophosphorylation that triggers phosphorylation of downstream signaling proteins [13]. In calvarial sutures, FGFs are secreted by osteoblasts at the differentiated edge of the bones; they activate receptors involved in both osteoprogenitor cell proliferation and function in the conversion of these cells into differentiated osteoblasts [12, 14–22]. Once the FGFR signaling system is established in sutures, long-term skull growth depends on the maintenance and balance between the formation of new bone and the proliferation of the osteoprogenitor cell population as a reservoir of potential new osteoblasts [2, 12].

Genetic mutations in the fibroblast growth factor receptors (FGFR1–3) are some of the most commonly identified mutations implicated in syndromic craniosynostosis [23–32]. Most of these mutations are autosomal dominant, gain-of-function mutations. The amplified signaling that results from these mutations plays an important role in the overossification at the site of sutures [2, 12, 21]. In addition to the FGFR signaling mutations, TWIST1 is implicated in craniosynostosis in humans with Saethre-Chotzen syndrome [33–36]. Mutations within TWIST1, a basic-helix-loop-helix transcription factor, result in TWIST1 haploinsufficiency, presenting as unilateral or bilateral coronal suture fusion among other facial malformations in patients with craniosynostosis [1, 33–35, 37]. TWIST1 is known to function as a regulator of mesenchymal lineage specification during skeletal development including within the cranial vault [37]. Twist1 heterozygous knockout mice have been shown to recapitulate the craniosynostosis phenotype of Saethre-Chotzen syndrome [38].

Previously, we have described a rabbit model with congenital nonsyndromic craniosynostosis of the coronal suture [39–44]. Similar to humans, this colony of affected New Zealand White rabbits demonstrates autosomal dominant transmission with variable phenotypic expression [39]. The animals present with a broad range of phenotypes for the isolated coronal suture synostosis pathology, including unilaterally or bilaterally affected animals that exhibit suture fusion at birth or with delayed-onset synostosis [41–44]. The genetic defect within this rabbit model is unknown, and a lack of molecular tools in rabbits has thus far made mapping genetic defects problematic. Herein, we conducted a molecular analysis of the rabbit colony to determine the cDNA coding sequence of Twist1 and the full-length sequence of FGFR3 (as the rabbit sequences of these genes were previously unknown). We used SNP analysis to determine whether FGFR1, FGFR2, or Twist1 were associated with the craniosynostosis phenotype of the rabbit colony.

## 2. Materials and Methods

### 2.1. Animals

All animal protocols were reviewed and approved by the Institutional Animal Care and Use Committee (IACUC). Animals were diagnosed and surgeries performed as previously described [45]. A 5 mm ear punch biopsy was obtained from 22 CS rabbits postmortem; the resulting biopsies were stored in RNAlater (Ambion, Austin, TX, USA) for use in genomic DNA extractions.

### 2.2. RNA Extraction/Purification of Samples/Preparation of cDNAs

For all WT and CS tissue RNA purifications, total RNA was purified from perisutural calvarial tissue of 10-day-old rabbits using the RNeasy Mini Kit (Qiagen Inc., Valencia, CA, USA) following manufacturer protocols after homogenization using a homogenizer and an on-column DNase treatment step as previously described [45]. Quality of RNA extracted was determined by capillary electrophoresis using an Agilent (Santa Clara, CA, USA) 2100 Bioanalyzer as previously described [45]. 

RNA from 2 WT and 2 CS rabbits was individually subjected to reverse transcription using SMARTScribe RT (Clontech, Mountain View, CA, USA) following manufacturer's protocol and utilizing the GeneRacer oligo dT reverse primer (Invitrogen, Carlsbad, CA, USA) and SMARTer II oligo (Clontech) for reverse transcription of all cDNAs for cloning.

### 2.3. Identification of Rabbit FGFR3/Twist1 cDNA Sequences

To determine rabbit sequence for FGFR3 and Twist1, primers were initially designed based on the predicted coding sequence for FGFR3 in Ochotona princeps (ENSOPRG00000002205) from Ensembl (http://www.ensembl.org/) and using human TWIST1 from Ensembl (ENST00000242261). For FGFR3, Ochotona princeps (American Pika) was chosen as it is a closely related mammal to rabbit. Various primer sets were designed using Vector NTI (Invitrogen) to amplify the FGFR3 rabbit cDNAs in a stepwise manner based on sequence similarity with FGFR3 in Ochotona princeps. Using these sequence fragments, a full-length rabbit cDNA sequence was determined from one WT rabbit RNA sample (data not shown). This sequence is given as Supplemental Figure 3  of the Supplementary Material available online at http://dx.doi.org/10.1155/2013/305971 and was used as a baseline to determine whether FGFR3 was structurally mutated in the CS animals. 

Primers T1-1 and T1-2, matching human TWIST1, were used to amplify the rabbit coding sequence of Twist1 from one WT rabbit. PCR was done using the AccuPrime HF system with buffer no. 1 (Invitrogen) supplemented with 2X PCRx Enhancer (Invitrogen), using the following cycling parameters: 95°C for 2 minutes, 43 cycles of 95°C for 25 seconds, 62.5°C for 30 seconds, 68°C for 1 minutes, on an MJ Tetrad cycler (Bio-Rad, Hercules, CA, USA). PCR reactions were separated on 1% TAE agarose gels, and the resulting primary amplicon of ~600 bp was gel extracted and sequenced. The rabbit Twist1 coding region consisted of 627 bp, and an alignment of human to rabbit Twist1 is given as Supplemental Figure 1.

### 2.4. Amplification of the FGFR3 cDNA Coding Region

Prepared cDNAs from WT and CS animals were used to clone FGFR3 in three steps; first, the majority of the coding region plus the 3′ UTR were obtained using primers F3–1 and F3-2. 5′ RACE was performed utilizing the SMARTer PCR cDNA synthesis system following the manufacturer instructions (Clontech). Clones consisting of the 5′ UTR and an overlapping part of the coding sequence were obtained first using primers F3-3 with the Clontech Universal Primer Mix. Clones consisting of the terminal 3′ UTR were obtained using primers F3–5 with the GeneRacer 3R Primer. Nested PCR was performed to enrich for 5′ and 3′ UTR target specific PCR products using 1 *μ*L purified PCR aliquot from the first round of PCR, the Clontech nested 5′ primer or GeneRacer Nested 3′ primer, and primer F3-4 (5′ UTR) or primer F3–6 (3′ UTR). All PCRs were done using the AccuPrime HF system with buffer no. 1 (Invitrogen). PCR reactions were separated on 0.8% TAE agarose gels, and the resulting primary amplicons were gel extracted following standard protocols (QIAquick Spin kit, Qiagen). 

The resulting cDNA PCR amplimers were subcloned into the pCR4 vector with TOPO-mediated cloning and transformed into TOP10 cells by electroporation as previously described [33]. Eight clones were selected for each rabbit cDNA; cloned vectors were propagated and miniprepped as previously described [33]. Plasmids were sequenced using M13 forward, M13 reverse, and various custom primers on an Applied Biosystems (Foster City, CA, USA) 3730xl DNA Analyzer using standard methods. All sequences were compared to the identified rabbit sequence for FGFR3 using the basic local alignment search tool “BLAST” program (http://www.ncbi.nlm.nih.gov/BLAST/).

### 2.5. Genotyping SNPs for FGFR1, FGFR2, and Twist1

Genomic DNA from CS ear punch biopsies was purified using the tissue lysis protocol for the DNeasy genomic DNA purification system (Qiagen). Genomic DNA was eluted in AE buffer and quantified using an ND-1000 spectrophotometer (NanoDrop Technologies, Inc., Wilmington, Del, USA).

To identify single nucleotide polymorphisms, we first designed primers using Vector NTI (Invitrogen) based on the predicted coding sequence for FGFR1 in rabbit (ENSOPRG00000002205) from Ensembl (http://www.ensembl.org/) and the published rabbit FGFR2 sequence from NCBI (http://www.ncbi.nlm.nih.gov/) [46]. Genomic PCR was performed using the AccuPrime HF system with buffer no. 2 (Invitrogen) and Platinum Taq Polymerase using primers F1-e9-SNPF and F1-e9-SNPR (FGFR1) and F2-SNPF and F2-SNPR (FGFR2); 5% DMSO (final concentration) was added to FGFR1 genotyping reactions. The following cycling parameters were used for genomic DNA amplification: 95°C for 2 minutes, 45 cycles of 95°C for 30 seconds, 58.7°C for 30 seconds, 68°C for 1.5 minutes, on an MJ Tetrad cycler (Bio-Rad); 61°C annealing temperature was used for FGFR1 instead of 58.7°C. PCR reactions were originally examined on a 1% TAE agarose gel, yielding single amplicons of 1472 bp (FGFR1) and 930 bp (FGFR2). PCR reactions were purified using the Nucleospin96 system following the manufacturer's protocol (Macherey-Nagel, Inc., Bethlehem, Pa, USA) with elution in 30 *μ*L 5 mM Tris, pH 8.5. Purified PCR products were directly sequenced with the sequencing primers F1-e9-SNP-seqF (FGFR1) or F2-SNP-seqF (FGFR2) on an Applied Biosystems (Foster City, CA, USA) 3730xl DNA Analyzer using standard methods. Resulting sequencing data were aligned in Sequencher (Gene Codes Corp., Ann Arbor, MI, USA) to identify SNPs.

Several single nucleotide polymorphisms (SNPs) were identified while obtaining the rabbit coding sequence for Twist1. Genomic PCR was performed utilizing the AccuPrime HF system with buffer no. 2 (Invitrogen), PCRx Enhancer (Invitrogen, 2 X final concentration), and Platinum Taq Polymerase using primers T1-1 and T1-2 (Table 1). The following cycling parameters were used for genomic DNA amplification: 95°C for 2 minutes, 45 cycles of 95°C for 30 seconds, 64°C for 30 seconds, 68°C for 1.5 minutes, on an MJ tetrad cycler (Bio-Rad). PCR reactions were originally verified on a 1% TAE agarose gel, yielding single amplicons of 627 bp (Twist1). PCR reactions were purified using the Nucleospin96 system following the manufacturer's protocol (Macherey-Nagel, Inc., Bethlehem, Pa, USA) with elution in 30 *μ*L 5 mM Tris, pH 8.5. Purified PCR products were directly sequenced utilizing standard sequencing conditions with the sequencing primer T1-SNP-seqF (Table 1). Resulting sequencing data were aligned in Sequencher (Gene Codes Corp., Ann Arbor, MI, USA) to identify SNPs.

## 3. Results

Our main objective was to determine whether Twist1, FGFR1, FGFR2, or FGFR3 were the sites of causative mutation within our rabbit model of craniosynostosis. Because the Twist1 sequence in rabbit was unavailable, we initially cloned and sequenced the coding portion of the cDNA for Twist1 from a WT rabbit, obtaining novel Twist1 coding DNA sequence. The structural coding sequence of rabbit Twist1 cDNA is 627 bp long as compared to the 609 bp TWIST1 coding sequence in humans (NCBI, NM_000474); Supplemental Figures  1  and  2  present the alignments of the Twist1 cDNA and protein homologs. Although we did not clone the full cDNA sequence, we identified several SNPs within the rabbit Twist1 cDNA sequence that made full sequencing unnecessary for our purposes, as described below. All of the identified SNPs were silent mutations.

**Figure 1:**
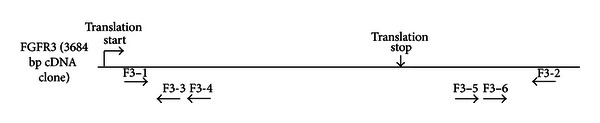
No structural mutations in FGFR3 in CS rabbits. Schematic depiction of FGFR3, with locations of primers employed. Full-length rabbit clones for FGFR3 were 3684 bp, as compared to approximately 4300 bp in human FGFR3.

The rabbit sequence for FGFR1 was available from Ensembl, and we previously published the rabbit sequence for FGFR2 [46]. However, to address FGFR3, we initially cloned and sequenced the entire cDNA for FGFR3 from WT rabbit RNA template, obtaining novel full-length FGFR3 cDNA sequence including 5′ and 3′ UTR sequences. Using the identified FGFR3 cDNA sequence, we purified RNA from calvariae of 10-day-old rabbits, using two WT and two CS rabbits, and we cloned and sequenced FGFR3 from at least eight different clones in three steps as described in Section 2 (Figure 1). The sequences obtained matched the rabbit coding sequence we originally identified for WT FGFR3. Rabbit FGFR3 was determined to be 3684 bp long, resulting in a predicted protein of 802 amino acids, as compared to the 4304 bp FGFR3 sequence in humans (NCBI, NM_000142.4) that results in a predicted protein of 806 amino acids; Supplemental Figures  3  and  4  consist of alignments between the FGFR3 cDNA and protein homologs. Multiple isoforms of FGFR3 are known in humans, and several were identified in our rabbit samples as well, including two different 5′ UTRs and two different 3′ UTRs. However, there were no differences observed between the WT and CS animals in the types of isoforms present or in their sequences. 

**Figure 2:**
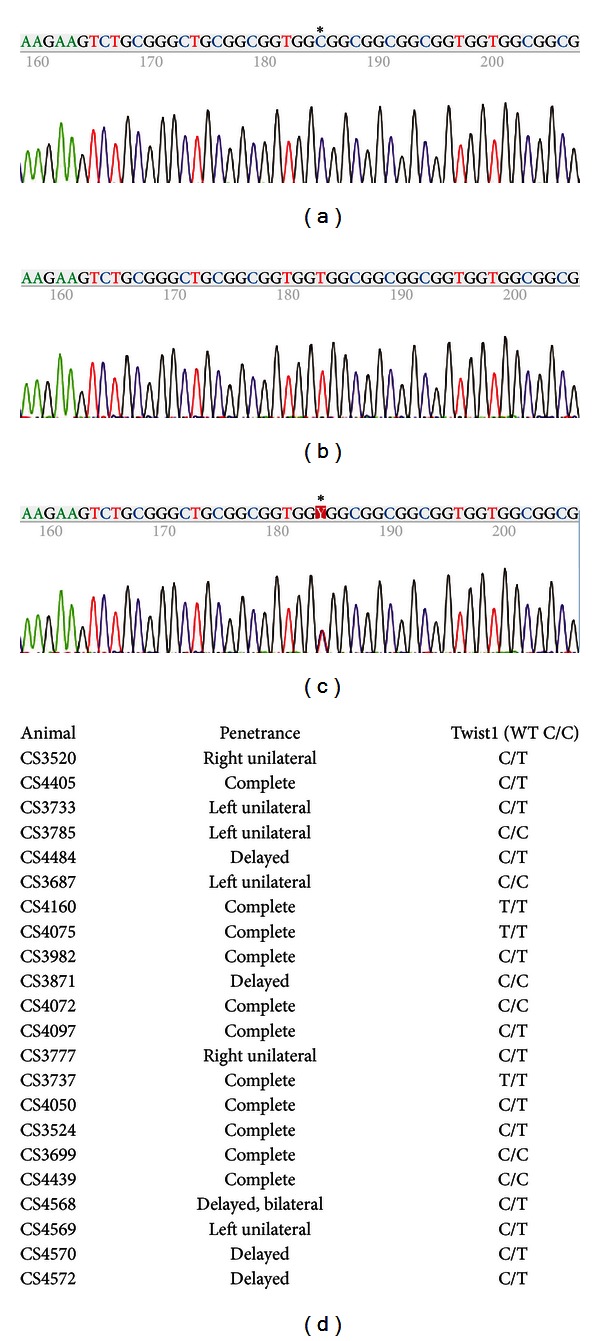
SNP discovery and genotyping of Twist1 in craniosynostotic rabbits. We identified a SNP within the Twist1 coding region, a silent mutation, that segregates independently of craniosynostosis within our colony, ruling out Twist1 as the causative agent. ((a)–(c)) Sequencing traces representing C/C (a), T/T (b), or C/T (c) genotypes from PCR using CS genomic DNA. (d) Results of sequencing 22 randomly selected CS animals; six animals were C/C genotype, three animals were T/T, and 13 animals were C/T.

Although no structural mutation within the coding sequence had been identified, we sought to more conclusively rule out the involvement of Twist1 within our rabbit model of craniosynostosis as well. We identified silent single nucleotide mutations within the coding sequence for Twist1 while cloning the rabbit Twist1 cDNA. SNP analysis on genomic DNA from 22 CS animals was then performed by sequencing PCR amplimers using primers flanking the chosen SNP. The SNP was identified as a variation within our animals of C/C, T/T, or C/T (Figures 2(a)–2(c)). Results of sequencing our 22 animals are reported in Figure 2(d); six animals were C/C, three animals were T/T, while 13 animals were C/T. These data indicated that it is extremely unlikely that Twist1 is the etiological locus of our craniosynostotic phenotype.

**Figure 3:**
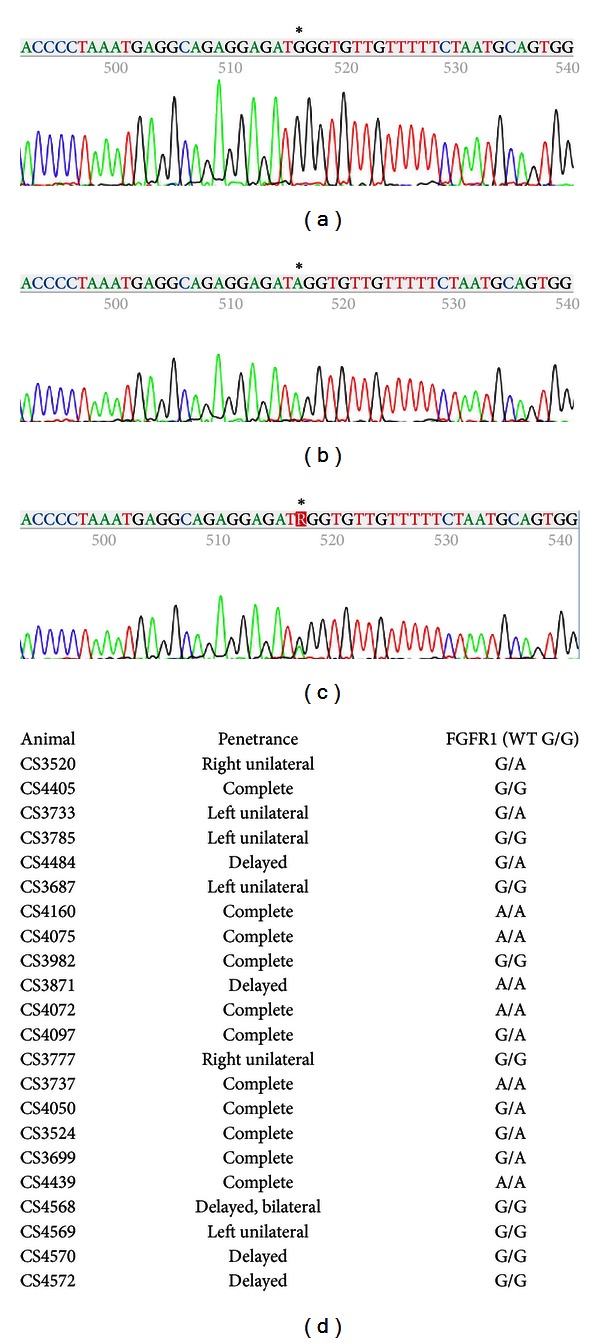
SNP discovery and genotyping of FGFR1 in craniosynostotic rabbits. We identified a FGFR1 SNP within intron 8 that segregates independently of craniosynostosis within our colony, ruling out FGFR1 as the causative agent. ((a)–(c)) Sequencing traces representing G/G (a), A/A (b), or G/A (c) genotypes from CS genomic DNA. (d) Results of sequencing of 22 randomly selected CS animals; nine animals were G/G, six animals were A/A, and seven animals were G/A.

We next sought to similarly determine whether FGFR loci were linked to the inherited craniosynostosis observed in our colony by using independent assortment of SNPs. We sequenced across exons 8 and 9 within FGFR1, including the splice sites flanking both exons. Amplified PCR products from genomic DNA obtained from 22 randomly selected yet related CS animals from the craniosynostotic colony made up our test group. There were no mutations within the exons (data not shown), but we identified a mutation within intron 8. This SNP was originally reported as “G” at Ensembl, while our animals variously carry G/G, A/A, or G/A (Figures 3(a)–3(c)). Results of sequencing our 22 CS animals are reported in Figure 3(d); nine animals were G/G, six animals were A/A, and seven animals were G/A. Thus, neither the “G” nor the “A” allele cosegregated with the disease phenotype, and a plurality of affected animals actually shared the G/G genotype found in wild type rabbit sequence as reported in Ensembl. 

**Figure 4:**
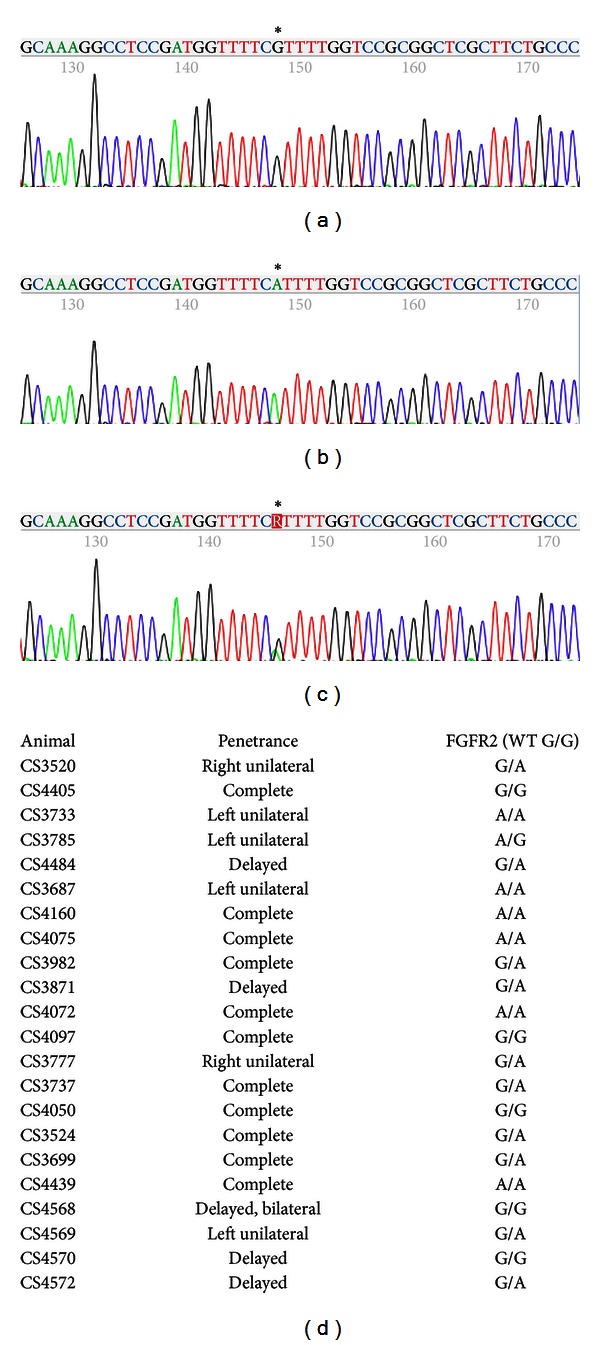
SNP discovery and genotyping of FGFR2 in craniosynostotic rabbits. We identified a FGFR2 SNP within exon 9, a silent mutation, that segregates independently of craniosynostosis within our colony, ruling out FGFR2 as the causative agent. ((a)–(c)) Sequencing traces representing G/G (a), A/A (b), or G/A (c) genotypes from CS genomic DNA. (d) Results of sequencing of 22 randomly selected CS animals; five animals were G/G genotype, seven animals were A/A, and ten animals were G/A.

For FGFR2, after sequencing across exon 9 including the flanking splice sites, we identified an SNP, a structurally silent mutation, within the coding sequence. This SNP was originally reported as “G” at NCBI, while our animals display G/G, A/A, or G/A (Figures 4(a)–4(c)). Results of sequencing from our 22 CS animals are reported in Figure 4(d); five animals were G/G genotype, seven animals were A/A, and ten animals were G/A. As with FGFR1, neither the “G” nor the “A” allele cosegregates with the affected phenotype, rendering it extremely unlikely that this locus is the site of causative mutation. 

## 4. Discussion

TWIST1 mutations were some of the first genetic defects linked to craniosynostosis in humans [27–29]. In addition, mutations within FGFR1–3 are known to cause craniosynostosis in humans [1–3, 15]. The sequences of FGFR3 and Twist1 (mRNA and gene) were previously unknown in rabbits. We have now determined the sequence of FGFR3 cDNA in the rabbit and determined that no structural mutations are present to explain the craniosynostotic phenotype, such as the P250R mutation commonly observed in Muenke syndrome [25]. We did identify several splice variants within FGFR3, including transcripts containing and lacking the “VT” amino acid insertion at the end of exon 10; however, these have been previously studied in other animals and in other FGFRs and were present within both WT and CS populations [47–49]. All splice variants obtained would result in the same predicted protein of 802 amino acids (or 804 if containing the additional “VT” amino acids described above). Also identified were two different 5′ UTRs and two different 3′ UTRs; however, these variants were also present in both WT and CS animals. These variants comprised a minor subset of the transcripts detected. The predominant transcript of FGFR3 corresponded to the full-length clone presented and was identical between WT and CS animals. No mutations were detected exclusively in CS FGFR3 transcripts. In addition, we have determined the Twist1 coding sequence in rabbit. Our one WT rabbit contained only the 627 bp form of Twist1, as did all mutant animals tested. Previously, it has been reported in humans that the glycine tract of TWIST1 in humans can have various numbers of polyglycine repeats [50]. It is possible that these rabbits may also have a variable number of polyglycine repeats, but we did not specifically examine for this, as our SNP analysis of Twist1 had already ruled it out as the causative locus. 

Extensive studies within this rabbit colony have shown that the CS phenotype is inherited in an autosomal dominant manner [51]. We used genomic DNA obtained from inbred, randomly selected CS rabbits to determine whether the SNPs we identified in FGFR1, FGFR2, and Twist1 were linked to the presence of craniosynostosis. Our data show that the identified SNPs in FGFR1, FGFR2, and Twist1 all segregated independently of the craniosynostotic phenotype, and therefore a mutation within these genes is highly unlikely to be the causative agent of the craniosynostosis within our model. In addition, independent analysis of FGFR2 within this colony identified another SNP that also failed to segregate with the craniosynostotic phenotype (data not shown), further supporting our results.

These genes play roles in numerous syndromes that involve craniosynostosis, including Apert, Crouzon, Pfeiffer, and Jackson-Weiss syndromes [23–32], but each of these syndromes contains multiple malformations in addition to fusion of cranial sutures. This rabbit colony, however, does not present with syndromic craniosynostosis but rather simple craniosynostosis, with isolated fusion of primarily the coronal suture(s). Previously we have reported that animals have been identified within the colony that exhibit fusion of the metopic suture; however, we believe that these fusion events are likely the result of a multifactorial process and these animals exhibit 100% mortality [52]. Therefore, our studies described herein focused on the molecular characterization of rabbits containing confirmed phenotypes of either wild type or coronal simple craniosynostosis. We previously reported that FGFR2 did not have any mutations detected across the coding sequence within these animals including the mutations that have been observed in human syndromes [46]. The current study indicates that this rabbit model of nonsyndromic craniosynostosis likely does not devolve from any other mutation outside of the FGFR2 coding region and extends this same conclusion to FGFR1 and Twist1.

In conclusion, we sought to determine whether either FGFR1–FGFR3 or Twist1 was the locus of the defect resulting in our rabbit heritable model of craniosynostosis. We provide novel rabbit FGFR3 and Twist1 sequence data and detected no obvious mutations comparing WT and mutant animals; through SNP analysis, we also eliminate FGFR1, FGFR2, and Twist1 as the etiological source of our defect. This is not surprising as most mutations in humans that cause craniosynostosis (including mutations in FGFR1–3 and TWIST1) tend to occur in syndromic cases of craniosynostosis, although some reports have described clinical cases where mutations in FGFR3 or TWIST1 are identified in nonsyndromic craniosynostosis [53]. We have recently determined that Tgf*β* receptors 1 and 2 are also unlikely to be the loci of origin in this rabbit model of craniosynostosis [54]. In addition, using SNP analysis, MSX2 does not appear to be the causative agent of craniosynostosis in these rabbits [55]; this was expected as gain-of-function mutations in MSX2 were identified primarily in one family and result in Boston type craniosynostosis in humans [56]. This suggests that these animals may contain a mutation of an as yet unknown effector of one of the major pathways involved in craniosynostosis, which may possibly shed light on the 85% of human craniosynostosis that are currently of an unknown origin. Of recent interest, TCF12 has been implicated in human nonsyndromic craniosynostosis, and we intend to investigate its role in this colony as well [57]. Work within this rabbit model has thus far been hampered due to the lack of readily available molecular tools in the rabbit. Recent advances in next generation sequencing may provide a method to rapidly genotype animals where detailed genomic data does not yet exist, providing an avenue to identify the etiology of craniosynostosis in these animals [58].

## Supplementary Material

Supplemental Figure 1: Alignment of rabbit and human TWIST1 cDNA The previously unknown rabbit clone representing the coding sequence of TWIST1 was aligned to human TWIST1 (NCBI, NM_000474) using ClustalO pairwise alignment. The structural coding sequence of rabbit Twist1 cDNA is 627 bp long as compared to the 609 bp TWIST1 coding sequence in humans.
Supplemental Figure 2: Alignment of rabbit, mouse, and human TWIST1 protein The predicted rabbit TWIST1 protein was aligned to mouse TWIST1 (NCBI, NP_035788.1) and human TWIST1 (NCBI, NP_000465.1) using ClustalO pairwise alignment. Rabbit TWIST1 was predicted to produce a protein of 209 amino acids, as compared to the 206 amino acid mouse TWIST1 and 202 amino acid human TWIST1. All structural regions of the protein are conserved between the various species with the majority of variation occurring within the poly-glycine tract.
Supplemental Figure 3: Alignment of rabbit and human FGFR3 cDNA Full-length rabbit clone of FGFR3 was aligned to human FGFR3 (NCBI, NM_000142.4) using ClustalO pairwise alignment. Rabbit FGFR3 was determined to be 3684 bp long as compared to the 4304 bp FGFR3 sequence in humans.
Supplemental Figure 4: Alignment of rabbit, mouse and human FGFR3 protein The predicted rabbit FGFR3 protein was aligned to mouse FGFR3 (NCBI, AAH53056.1) and human FGFR3 (NCBI, NP_000133.1) using ClustalO pairwise alignment. Rabbit FGFR3 was predicted to produce a protein of 802 amino acids, as compared to the 800 amino acid mouse FGFR3 and 806 amino acid human FGFR3. All structural regions of the protein are conserved between the various species.

## Figures and Tables

**Table 1:** Primers used in this study.

		Primer sequence

FGFR1 primers		
F1-e9-SNPF	F	TGAAACCAAACAGCCACCTACTG
F1-e9-SNPR	R	CGAAGACAGGACGACGATGAAAAC
F1-e9-SNP-seqF	F	TGGTGACGGGTCGTAAC
FGFR2 primers		
F2-SNPF	F	CCAGCCCATTTTTCACCGAAC
F2-SNPR	R	GAATCTCACTCCCACTGACATCATC
F2-SNP-seqF	F	GTCGTCAAGTGGTTATCCC
Twist1 primers		
T1-1	F	ATGATGCAGGACGTGTCCA
T1-2	R	CTAGTGGGACGCGGACATG
T1-SNP-seqF	F	ACGACAGCCTGAGCAACAG
FGFR3 primers		
F3–1	F	CGACAGGAACAGGTGGTCTTTGG
F3-2	R	CGGCACAGGCAGCAATTAAAG
F3-3	R	CCACCTTGCTGCCGTTCACCTC
F3-4	R	TCCACTGCTGGTGCCGCAGCTTG
F3–5	F	GACGACTCCGTGTTCACCC
F3–6	F	GGAGGGGACCAGAAGTAGAATGTAG
