# Supplementary material for: Molecular Analysis of Twist1 and FGF Receptors in a Rabbit Model of Craniosynostosis: Likely Exclusion as the Loci of Origin
Source: Int J Genomics. 2013 May 8;2013:305971. doi: 10.1155/2013/305971 (PMC3664496; doi:10.1155/2013/305971)
Supplement: Supplementary file 1 — Supplemental Figure 1: Alignment of rabbit and human TWIST1 cDNA The previously unknown rabbit clone representing the coding sequence of TWIST1 was aligned to human TWIST1 (NCBI, NM_000474) using ClustalO pairwise alignment. The structural coding sequence of rabbit Twist1 cDNA is 627 bp long as compared to the 609 bp TWIST1 coding sequence in humans. Supplemental Figure 2: Alignment of rabbit, mouse, and human TWIST1 protein The predicted rabbit TWIST1 protein was aligned to mouse TWIST1 (NCBI, NP_035788.1) and human TWIST1 (NCBI, NP_000465.1) using ClustalO pairwise alignment. Rabbit TWIST1 was predicted to produce a protein of 209 amino acids, as compared to the 206 amino acid mouse TWIST1 and 202 amino acid human TWIST1. All structural regions of the protein are conserved between the various species with the majority of variation occurring within the poly-glycine tract. Supplemental Figure 3: Alignment of rabbit and human FGFR3 cDNA Full-length rabbit clone of FGFR3 was aligned to human FGFR3 (NCBI, NM_000142.4) using ClustalO pairwise alignment. Rabbit FGFR3 was determined to be 3684 bp long as compared to the 4304 bp FGFR3 sequence in humans. Supplemental Figure 4: Alignment of rabbit, mouse and human FGFR3 protein The predicted rabbit FGFR3 protein was aligned to mouse FGFR3 (NCBI, AAH53056.1) and human FGFR3 (NCBI, NP_000133.1) using ClustalO pairwise alignment. Rabbit FGFR3 was predicted to produce a protein of 802 amino acids, as compared to the 800 amino acid mouse FGFR3 and 806 amino acid human FGFR3. All structural regions of the protein are conserved between the various species. [file 305971.f1.pdf]

CLUSTAL O(1.1.0) multiple sequence alignment

```
human           ATGATGCAGGACGTGTCCAGCTCGCCAGTCTCGCCGGCCGACGACAGCCTGAGCAACAGC
rabbit          ATGATGCAGGACGTGTCCAGCTCGCCAGTCTCGCCGGCCGACGACAGCCTGAGCAACAGC
                ************************************************************

human           GAGGAAGAGCCAGACCGGCAGCAGCCGCCGAGCGGCAAGCGCGGGGGACGCAAGCGGCGC
rabbit          GAGGAGGAGCCGGACCGGCAGCAGCCGCCGAGCGGCAAGCGCGGGGGGCGCAAGCGGCGC
                *****.*****.***********************************.************

human           AGCAGCAGGCGCAGCGCGGGCGGCGGCGCGGGGCCCGGCGGAGCCGCGGGTGGGGGCGTC
rabbit          AGCAGCCGGCGCAGCGCGGGCGGCGGCGCGGGGCCCGGCGGGGCCGCGGGCGGGGGCGTC
                ******.**********************************.******** *********

human           GGAGGCGGCGACGAGCCGGGCAGCCCGGCCCAGGGCAAGCGCGGCAAGAAGTCTGCGGGC
rabbit          GCAGCCGGCGATGAGCCCGGCAGCCCGGCGCAGGGCAAGCGCGGCAAGAAGTCTGCGGGC
                * ** ****** ***** *********** ******************************

human           TGTGGCGGCGGCGGCGGCGCG------------------GGCGGCGGCGGCGGCAGCAGC
rabbit          TGCGGCGGTGGCGGCGGCGGCGGTGGTGGCGGCGCGGGCGGCGGCGGTGGCGGCAGCAGC
                ** ***** **********                    ******** ************

human           AGCGGCGGCGGGAGTCCGCAGTCTTACGAGGAGCTGCAGACGCAGCGGGTCATGGCCAAC
rabbit          AGCGGCGGCGGGAGCCCGCAGTCGTACGAGGAGCTGCAGACGCAGCGGGTCATGGCCAAT
                ************** ******** *********************************** 

human           GTGCGGGAGCGCCAGCGCACCCAGTCGCTGAACGAGGCGTTCGCCGCGCTGCGGAAGATC
rabbit          GTGCGGGAGCGCCAGCGCACGCAGTCGCTGAACGAGGCGTTCGCCGCGCTGCGGAAGATC
                ******************** ***************************************

human           ATCCCCACGCTGCCCTCGGACAAGCTGAGCAAGATTCAGACCCTCAAGCTGGCGGCCAGG
rabbit          ATCCCCACGCTGCCTTCGGACAAGCTGAGCAAGATCCAGACGCTCAAGCTGGCCGCCAGG
                ************** ******************** ***** *********** ******

human           TACATCGACTTCCTCTACCAGGTCCTCCAGAGCGACGAGCTGGACTCCAAGATGGCAAGC
rabbit          TACATCGACTTCCTGTACCAGGTCCTGCAGAGCGACGAGCTGGACTCCAAGATGGCAAGC
                ************** *********** *********************************

human           TGCAGCTATGTGGCTCACGAGCGGCTCAGCTACGCCTTCTCGGTCTGGAGGATGGAGGGG
rabbit          TGCAGCTACGTGGCCCACGAGCGGCTCAGCTACGCCTTCTCGGTCTGGAGGATGGAGGGG
                ******** ***** *********************************************

human           GCCTGGTCCATGTCCGCGTCCCACTAG
rabbit          GCCTGGTCCATGTCCGCGTCCCACTAG
                ***************************
```

```
CLUSTAL O(1.1.0) multiple sequence alignment


human           MMQDVSSSPVSPADDSLSNSEEEPDRQQPPSGKRGGRKRRSSRRSAGGGAGPGGAAGGGV
mouse           MMQDVSSSPVSPADDSLSNSEEEPDRQQPASGKRGARKRRSSRRSAGGSAGPGGATGGGI
rabbit          MMQDVSSSPVSPADDSLSNSEEEPDRQQPPSGKRGGRKRRSSRRSAGGGAGPGGAAGGGV
                ***************************** *****.************.******:***:

human           GGGDEPGSPAQGKRGKKSAGCGGGGGAG------GGGGSSSGGGSPQSYEELQTQRVMAN
mouse           GGGDEPGSPAQGKRGKKSAGGGGGGGAGG--GGGGGGGSSSGGGSPQSYEELQTQRVMAN
rabbit          AAGDEPGSPAQGKRGKKSAGCGGGGGGGGGGAGGGGGGSSSGGGSPQSYEELQTQRVMAN
                ..****************** *****.*      **************************

human           VRERQRTQSLNEAFAALRKIIPTLPSDKLSKIQTLKLAARYIDFLYQVLQSDELDSKMAS
mouse           VRERQRTQSLNEAFAALRKIIPTLPSDKLSKIQTLKLAARYIDFLYQVLQSDELDSKMAS
rabbit          VRERQRTQSLNEAFAALRKIIPTLPSDKLSKIQTLKLAARYIDFLYQVLQSDELDSKMAS
                ************************************************************

human           CSYVAHERLSYAFSVWRMEGAWSMSASH
mouse           CSYVAHERLSYAFSVWRMEGAWSMSASH
rabbit          CSYVAHERLSYAFSVWRMEGAWSMSASH
                ****************************
```

```
rabbit      1                                   GCGGGCGGGGCGGCCGGGGGCCGGGGGCCGGAGGGCTCGGGGCTCAGGACCCTGGCCGCGGCCGCC   66
human       1 GTCGCGGGCAGCTGGCGCCGCGCGGTCCTGCTCTGCCGGTCGCACGGACGC--ACCGGCGGGCCGCCGGCCGGAGGGACGGGGCGGGAGCTGGGCCCGCG   98
                                                ** **  *  *** **    **** ** * *  ***  * **  * ** *    ** * * ****

rabbit     67 GCTGCCGCGC--GCCCCGGGGCCGGTGACGAGGTGCCGCCCTCCTCCTCCCGCAGACTCCGGCGTGCGCCTTCCTCCCTCTCGGGCCCCGGCCGGGC  164
human      99 GACAGCGAGCCGGAGCGGGAGCCGCGCGTAGCGAGCCGGGCTCCGGCGCTCGCCAGTCTCCCGAGCG-GCGCCCGCCTCCCGCCGGTGCCCGCGCCGGGC  197
              *    ** **  *  * ** ****        * ****  ****  * *   **     * *  *** *****  ****** *  * ****  *******

rabbit    165 CGTGGGGGGCAGCATGCCCGCGCGCGCCGCCTGAGGACGCCGG---CTCGGTCCCCGCCATGGGCGCCCCGGCCCGCGCGCTGGCACTCTGCGTGGCCGT  261
human     198 CGTGGGGGGCAGCATGCCCGCGCGCGCTGCCTGAGGACGCCGCGGCCCCCGCCCCCGCCATGGGCGCCCCTGCCTGCGCCCTCGCGCTCTGCGTGGCCGT  297
              *************************** **************    * * * ***************** *** **** ** ** **************

rabbit    262 GGTGGTCGTGGCCGGCGTCGTCTGGGAGCCTGCGGGTCCGGAGCGGCGTGTGGTGCGGAGAGCCGCAGGGGTGCCCGGGTTGGAGCCCAGCCGACAGGAA  361
human     298 GGCCATCGTGGCCGGCGCCTCCTCGGAGTCCTTGGGGACGGAGCAGCGCGTCGTGGGGCGAGCGGCAGAAGTCCCGGGCCCAGAGCCCGGCCAGCAGGAG  397
              **   *********** *  ** **** *   ***  ****** *** ** *** ** **** ****  ** ** **    ****** ***  *****

rabbit    362 CAGGTGGTCTTTGGCAGTGGGGACACCGTGGAGCTGAGCTGCCACCTTCCTGGAGGTGGCCCCCTGGGGCCCACCGTCTGGGTCAAGGACGGCACGGGGC  461
human     398 CAGTTGGTCTTCGGCAGCGGGGATGCTGTGGAGCTGAGCTGTCCCCCGCCCGGGGGTGGTCCCATGGGGCCCACTGTCTGGGTCAAGGATGGCACAGGGC  497
              *** ******* ***** *****  * ************** * **  ** ** ***** *** ********** ************** ***** ****

rabbit    462 TGGCGTCCACGGACCGCATCCTGGTCGGGCCTCAACGGCTGCAGGTGCTGAACGCCTCCCAGGAGGACGCGGGGGCCTACAGCTGCCGGCAGCGGCTCAC  561
human     498 TGGTGCCCTCGGAGCGTGTCCTGGTGGGGCCCCAGCGGCTGCAGGTGCTGAATGCCTCCCACGAGGACTCCGGGGCCTACAGCTGCCGGCAGCGGCTCAC  597
              *** * ** **** **  ******* ***** ** **************** ******** ****** * ****************************

rabbit    562 GCAGCGTGTGCTGTGCCACTTCATTGTGCGGCTGACCGAGGCTCCGTCCTCGGGAGATGACGAAGACGGCGAGGATGAGGCCGAAGACACAGG-------  654
human     598 GCAGCGCGTACTGTGCCACTTCAGTGTGCGGGTGACAGACGCTCCATCCTCGGGAGATGACGAAGACGGGGAGGACGAGGCTGAGGACACAGGTGTGGAC  697
              ****** ** ************* ******* **** ** ***** ********************** ***** ***** ** ********

rabbit    655 -----GGCCCCTTACTGGACCCGGCCCGAGCGGATGGACAAGAAGCTGCTGGCGGTGCCGGCCGCCAACACCGTCCGCTTCCGCTGCCCGGCCGCCGGCA  749
human     698 ACAGGGGCCCCTTACTGGACACGGCCCGAGCGGATGGACAAGAAGCTGCTGGCCGTGCCGGCCGCCAACACCGTCCGCTTCCGCTGCCCAGCCGCTGGCA  797
                   *************** ******************************** ********************************** ***** ****

rabbit    750 ACCCGACGCCCTCCATCTCCTGGCTGAAGAATGGCAAAGAGTTTCGCGGCGAGCACCGCATCGGCGGCATCAAGCTGCGGCACCAGCAGTGGAGCCTGGT  849
human     798 ACCCCACTCCCTCCATCTCCTGGCTGAAGAACGGCAGGGAGTTCCGCGGCGAGCACCGCATTGGAGGCATCAAGCTGCGGCATCAGCAGTGGAGCCTGGT  897
              **** ** ********************** ****  ***** ***************** ** ***************** *****************

rabbit    850 GATGGAGAGCGTGGTGCCATCGGACCGCGGCAACTACACGTGCGTGGTGGAGAACAAGTTCGGCAGCATCCGGCAGACGTACACGCTGGACGTGCTGGAG  949
human     898 CATGGAAAGCGTGGTGCCCTCGGACCGCGGCAACTACACCTGCGTCGTGGAGAACAAGTTTGGCAGCATCCGGCAGACGTACACGCTGGACGTGCTGGAG  997
               ***** *********** ******************* ***** ************** ****************************************

rabbit    950 CGCTCCCCGCACCGGCCCATCCTGCAGGCCGGGCTGCCCGCCAACCAGACGGCCGTGCTGGGCAGCGACGTGGAGTTCCACTGCAAGGTGTACAGCGACG 1049
human     998 CGCTCCCCGCACCGGCCCATCCTGCAGGCGGGGCTGCCGGCCAACCAGACGGCGGTGCTGGGCAGCGACGTGGAGTTCCACTGCAAGGTGTACAGTGACG 1097
              ***************************** ******** ************** ***************************************** ****

rabbit   1050 CGCAGCCGCACATCCAGTGGCTGAAGCACGTGGAGGTGAACGGCAGCAAGGTGGGCCCCGACGGCACGCCCTACGTCACCGTGCTGAAGACGGCGGGCGC 1149
human    1098 CACAGCCCCACATCCAGTGGCTCAAGCACGTGGAGGTGAATGGCAGCAAGGTGGGCCCGGACGGCACACCCTACGTTACCGTGCTCAAGACGGCGGGCGC 1197
              * ***** ************** ***************** ***************** ******** ******** ******** **************

rabbit   1150 TAACACCACCGACAAGGAGCTAGAGGTTCTGTCTTTGCGCAATGTCACCTTTGAGGATGCGGGGGAGTACACGTGCCTGGCGGGCAATTCTATCGGGTTT 1249
human    1198 TAACACCACCGACAAGGAGCTAGAGGTTCTCTCCTTGCACAACGTCACCTTTGAGGACGCCGGGGAGTACACCTGCCTGGCGGGCAATTCTATTGGGTTT 1297
              ****************************** ** **** *** ************** ** *********** ******************** ******

rabbit   1250 TCCCATCACTCTGCGTGGCTGGTGGTGCTGCCAGCCGAGGAGGAGCTGGCGGCGGCCGGCGAGGCGGGCAGCGTGTACGCGGGCGTCCTCAGCTACGGCG 1349
human    1298 TCTCATCACTCTGCGTGGCTGGTGGTGCTGCCAGCCGAGGAGGAGCTGGTGGAGGCTGACGAGGCGGGCAGTGTGTATGCAGGCATCCTCAGCTACGGGG 1397
              ** ********************************************** ** *** * ************ ***** ** *** ************* *

rabbit   1350 CGGGCTTCTTCCTCTTCATCCTGGTGGTGGCGCCGTGACGCTCTGCCGCCTGCACAGCCCCCCAAGAAGGGGCTGGGCTCCCCGACGGTGCACAAGGT 1449
human    1398 TGGGCTTCTTCCTGTTCATCCTGGTGGTGGCGGCTGTGACGCTCTGCCGCCTGCGCAGCCCCCCAAGAAAGGCCTGGGCTCCCCCACCGTGCACAAGAT 1497
               ************ ****************  ** ******************* *************** ** *********** ** ********* *

rabbit   1450 GTCCCGCTTCCCGCTGAAGCGCCAGGTGTCCTTGGAGTCCAGCTCGTCCATGAACTCCACGACGCCGCTGGTGCGCGTGGCGCGGCTGTCCTCCGCCGAG 1549
human    1498 CTCCCGCTTCCCGCTCAAGCGACAGGTGTCCCTGGAGTCCAACGCGTCCATGAGCTCCAACACACCACTGGTGCGCATCGCAAGGCTGTCCTCAGGGGAG 1597
               ************** ***** ********* ********* * ********* *****  ** ** ********* * **  ********** *  ***

rabbit   1550 GGGCCCACGCTGGCCAACGTGTCCGAGCTCGAGCTGCCCGCCGACCCGCAGTGGGAGCTGTCCCGGGCCCGGCTGACCCTCGGGAAGCCCCTGGGAGAGG 1649
human    1598 GGCCCCACGCTGGCCAATGTCTCCGAGCTCGAGCTGCCTGCCGACCCCAAATGGGAGCTGTCTCGGGCCCGGCTGACCCTGGGCAAGCCCCTTGGGGAGG 1697
              ** ************** ** ***************** ********  * *********** ***************** ** ******** ** ****

rabbit   1650 GCTGCTTCGGCCAGGTGGTCATGGCCGAGGCCATCGGCATCGACAAGGACCGGGCCGCCAAGCCCGTCACCGTGGCCGTGAAGATGCTGAAAGACGATGC 1749
human    1698 GCTGCTTCGGCCAGGTGGTCATGGCGGAGGCCATCGGCATTGACAAGGACCGGGCCGCCAAGCCTGTCACCGTAGCCGTGAAGATGCTGAAAGACGATGC 1797
              ************************* ************** *********************** ******** **************************

rabbit   1750 CACCGACAAAGACCTGTCGGACCTGGTGTCGGAGATGGAGATGATGAAGATGATCGGGAAGCACAAGAACATCATCAACCTGCTGGGCGCCTGCACGCAG 1849
human    1798 CACTGACAAGGACCTGTCGGACCTGGTGTCTGAGATGGAGATGATGAAGATGATCGGGAAACACAAAAACATCATCAACCTGCTGGGCGCCTGCACGCAG 1897
              *** ***** ******************** ***************************** ***** *********************************

rabbit   1850 GGCGGGCCCCTGTACGTGCTGGTGGAGTTCGCGGCCAAGGGCAACCTGCGGGAGTACCTGCGGGCGCGCCGGCCTCCGGGCATGGACTACTCGTTCGACA 1949
human    1898 GGCGGGCCCCTGTACGTGCTGGTGGAGTACGCGGCCAAGGGTAACCTGCGGGAGTTTCTGCGGGCGCGGCGGCCCCCGGGCCTGGACTACTCCTTCGACA 1997
              **************************** ************ *************  *********** ***** ****** ********** *******

rabbit   1950 CCTGCAGGCTGCCCGAGGAGCAGCTCACCTTCAAGGACCTGGTGTCCTGCGCCTACCAGGTGGCCCGGGGCATGGAGTACCTGGCCTCGCAGAAGTGCAT 2049
human    1998 CCTGCAAGCCGCCCGAGGAGCAGCTCACCTTCAAGGACCTGGTGTCCTGTGCCTACCAGGTGGCCCGGGGCATGGAGTACTTGGCCTCCCAGAAGTGCAT 2097
              ****** ** *************************************** ****************************** ******* **********

rabbit   2050 CCACAGGGACCTGGCCGCCCGCAACGTGCTGGTGACCGAGGACAACGTGATGAAGATCGCGGACTTCGGCCTGGCCCGCGACGTGCACAACCTCGACTAC 2149
human    2098 CCACAGGGACCTGGCTGCCCGCAATGTGCTGGTGACCGAGGACAACGTGATGAAGATCGCAGACTTCGGGCTGGCCCGGGACGTGCACAACCTCGACTAC 2197
              *************** ******** *********************************** ******** ******** ********************

rabbit   2150 TACAAGAAGACCACCAATGGCAGGCTGCCCGTGAAGTGGATGGCGCCCGAGGCCCTGTTTGACCGCGTCTACACCCACCAGAGCGATGTCTGGTCCTTCG 2249
human    2198 TACAAGAAGACAACCAACGGCCGGCTGCCCGTGAAGTGGATGGCGCCTGAGGCCTTGTTTGACCGAGTCTACACTCACCAGAGTGACGTCTGGTCCTTTG 2297
              *********** ***** *** ************************* ****** ********** ******** ******** ** *********** *

rabbit   2250 GGGTCCTGCTTTGGGAGATCTTCACGCTGGGGGGCTCGCCGTACCCCGGCATCCTGTGGAGGAACTGTTCAAGCTGCTGAGAGAGGGCCACCGCATGGA 2349
human    2298 GGGTCCTGCTCTGGGAGATCTTCACGCTGGGGGGCTCCCCGTACCCCGGCATCCTGTGGAGGAGCTCTTCAAGCTGCTGAAGGAGGGCCACCGCATGGA 2397
              ********** ************************** ************************** ** *************  ****************

rabbit   2350 CAAGCCGGCCAACTGCACGCACGACCTGTACATGATCATGCGGGAGTGCTGGCACGCGGCGCCCTCGCAGAGGCCCACCTTCAAGCAGCTCGTGGAGGAC 2449
human    2398 CAAGCCCGCCAACTGCACACACGACCTGTACATGATCATGCGGGAGTGCTGGCATGCCGCGCCCTCCCAGAGGCCCACCTTCAAGCAGCTGGTGGAGGAC 2497
              ****** *********** *********************************** ** ********* ********************** *********
```

```
rabbit  2450 CTGGACCGCGTCCTCACCGTGACGTCCACTGACGAGTACCTGGACCTGTCGGCGCCCTTCGAGCAGTACTCCCCGGGCGGCCAGGACAGCCCCAGCGCCA 2549
human   2498 CTGGACCGTGTCCTTACCGTGACGTCCACCGACGAGTACCTGGACCTGTCGGCGCCTTTCGAGCAGTACTCCCCGGGTGGCCAGGACACCCCCAGCTCCA 2597
             ******** ***** ************** ************************** ******************** ********** ******* ***

rabbit  2550 GCTCCTCCGGGGACGACTCCGTGTTCACCCACGACCTGCTGCCCCCCGCGCCCCCCAGCAGCGGGGGCCCGCGGACTTGAAGGGCCGGGCGCCCC----- 2644
human   2598 GCTCCTCAGGGGACGACTCCGTGTTTGCCCACGACCTGCTGCCCCCGGCCCCACCCAGCAGTGGGGGCTCGCGGACGTGAAGGGCCACTGGTCCCCAACA 2697
             ******* *****************  ******************* ** ** ******** ****** ******* *********    * ***

rabbit  2645 -------AGGCCTGTGAGAGCCCACCCTGCCGCCGCTGCCCAGTG---CCCTGGCCTCGG-----GTGCGCGTGCGTGTGC----------------GT 2712
human   2698 ATGTGAGGGGTCCCTAGCAGCCCACCCTGCTGCTGGTGCACAGCCACTCCCCGGCATGAGACTCAGTGCAGATGGAGAGACAGCTACACAGAGCTTTGGT 2797
                     ** *  *   ************ ** * *** ***     *** *** *  *     ****   **      *                 **

rabbit  2713 GTGTGCGCGCGTGTGTGCGTGTGTGGCTGTCCCGAGGC-CGCGGACAAGGACAGCAGCACGGAGGGGACCAGAAGTAGAATGTAGGGG-ACGCCCCCCAC 2810
human   2798 CTGTGTGTGTGTGTGTGCGTGTGTGTGTGTGTGTGCACATCCGCGTGTGCCTGTGTGCGTGCGCATCTTGCCTCCAGGTGCAGAGGTACCCTGGGTGT 2897
              **** * * **************   ***      ** *     *  *  *    *  **   *   *  *        ** ** ** ** *

rabbit  2811 CCCCGCCGCCAGACCCCTGGGAGGGAGGAGCGGCCGGGCCCGAGCTGAGCCTGCCCTGCCCGGGGGCTCCGAGCCAGATTACGGGTACCTGAGGGCGGCA 2910
human   2898 CCCCGCTGCTGTGCAACGGTCTCCTGACTGGTGCTGCAGCACCGAGGGGCCT-TTGTTCTGGGGGGACCCAGTGCAGAATGTAAGTGGGCCCACCCGGTG 2996
             ****** **    *  * *          *  ** *   *   *  * ****    * *  *****  **    **** *    **         ***

rabbit  2911 GCCTGGCCTCTGCCCGGCAGGCTTCTTCCAGACGCGCGCGAGTGTGGACCTCCGCGTCCGGGAGGAGGGGGCCGCTGGCGCCGCCCACGCAGAGGCCCGC 3010
human   2997 G---GACCCCCGTGGGGCAGGGAGCT---GGGCCCGACATGGCTCCGGCCTCTGCCTTTGCACCACGGGACATCACAGGGTGGGCCTCGGCCCCTCCCAC 3090
             *   * ** * *   ******   **    * * **     *    * **** ** *  *      ***        * *  * ** **      *** *

rabbit  3011 GGGGCGCGAGGGATTTCCTGGCACTGCAGTTTTGTTCTAAAAATTGTACCTGGACCTGGATATTTATCGGCCCCCCAGACCCCCGGCCCCGCCCCGGCCC 3110
human   3091 ACCCAAAGCTGAGCCTGCAGGGAAGCCCCACATGTCCAGCACCTTGTGCCTGGGGTGTTAGTGGCACCGCCTCCCCA--CCTCCAGGCTTTCCCACTTCC 3188
                    *  *    * * ** *   *     *** *   *  **** *****      *     * ** * *****  ** ** * *   ***    **

rabbit  3111 CTTTTAACTTATTCACAGCCGAGCCGGCCGAGGCCCGGCGCTCGCCGCGGAGGTGGAGCCGTGCGGACGGCTCGTTCC------CCTCGGCCGTCCCTGG 3204
human   3189 CACCCTGCCCCTCAGAGACTGAAATTACGGGTACCTGAAGATGGGAGCCTTTACCTT-TTATGCAAAAGGTTTATTCCGGAAACTAGTGTACATTTCTAT 3287
             *      *   *      * **     * *   ** *  * * *  **             ***  * ** *  ****          *  * *  **

rabbit  3205 CGGGAAATGAGATTTCTGGAGGGGATTTGGTTTTTTGACTTCAAGGCAAGCTGGT-ATTTCCACACCAACCCTGCTTCTAATTGCTGCCTGTGCCGGGCG 3303
human   3288 AAATAGATGCTGTGTATATGGTATATATACATATATATATATAACATATATGGAAGAGGAAAAGGCTGGTACAACGGAGGCCTGCGACCCTGGGGGCACA 3387
                 * ***   * * *   *   ** *   * * *   *  **   *    *   *     *  *     *  *       ***  **   *  *  *

rabbit  3304 GGGCGGGGAGCCGGCCCTGCGTGCAGGCTC------CACGTTGTCAGATGTTACAAGTTTATATCTATCTATATCTATAATTTATTGCGTTTTTA-CAAG 3396
human   3388 GGAGGCAGGCATGGCCCTGGGCGGGGCGTGGGGGGGCGTGGAGGGAGGCCCCAGGGGTCTCACCCATGCAAGCAGAGGACCAGGGCCTTTTCTGGCACC 3487
             **  *  *    ******* * *  *  *       *  *  *  **     *   * *   * * **  *     *  *       * *** *  **

rabbit  3397 GTGCATTTGCCGTAGACTTAACACTTCTTGC--GCAACGCT---TCTAGACTTTTACAGCCTGGGCTGCTACTTTTCAAAGCTTGGGAGTGAAAGCTGCC 3491
human   3488 GCAGTTTTGTTTTAAAACTGGACCTGTATATTTGTAAAGCTATTTATGGGCCCCTGGCACTCTTGTTCCCACACCCCAACACTTCCAGCATTTAGCTGGC 3587
             *    ****   ** *  *    **   *    * ** ***   * * * *   *    *    * * * **    ***  ***         ***** *

rabbit  3492 -----GGTCGAGTTTCTTCCTTTTGTACTGTTTGGGTGGCCGGCTGTCCCTGCACGGCCAGCGGCGGCCAGCGGGCGGGGCCCAGTGGCCCAGTGGGCAG 3586
human   3588 CACATGGCGGAGAGTTTTAATTTTTAACTTATTGACAACCGAGAAGGTTTATCCCGCCGATAGAGGGACGGCCAAGAATGTACGTCCAGCCTGCCC-CGG 3686
                  **  ***  * **  ****  ***  ***     *  *  *      * ** * *  *  ** * **       *  *      ** *    * *

rabbit  3587 GGCTGCCAGGCCCCGGTGGTGGGTGTGGCTGTGTTAACCCAGCCGACGGACTTTCCAGAACATAAAGACGACCGGTTCCGAAAAAAAAAAAAAAAAAA   3684
human   3687 AGCTGGAGGATCCCCTCCAAGCCTAAAAGGTTGTTAAT--AGTTGGAGGTGATTCCAGTGAAGATATTTTATTTCCTTTGTCCTTTTTCAGGAGAATTAG 3784
              ****   *  ***      *  *       ******   **  *  **   ******   * * *    *     *  *         *  * **

rabbit  3685                                                                                                      3684
human   3785 ATTTCTATAGGATTTTTCTTTAGGAGATTTATTTTTTGGACTTCAAAGCAAGCTGGTATTTTCATACAAATTCTTCTAATTGCTGTGTGTCCCAGGCAGG 3884

rabbit  3685                                                                                                      3684
human   3885 GAGACGGTTTCCAGGGAGGGGCCGGCCCTGTGTGCAGGTTCCGATGTTATTAGATGTTACAAGTTTATATATATCTATATATATAATTTATTGAGTTTTT 3984

rabbit  3685                                                                                                      3684
human   3985 ACAAGATGTATTTGTTGTAGACTTAACACTTCTTACGCAATGCTTCTAGAGTTTTATAGCCTGGACTGCTACCTTTCAAAGCTTGGAGGGAAGCCGTGAA 4084

rabbit  3685                                                                                                      3684
human   4085 TTCAGTTGGTTCGTTCTGTACTGTTACTGGGCCCTGAGTCTGGGCAGCTGTCCCTTGCTTGCCTGCAGGGCCATGGCTCAGGGTGGTCTCTTCTTGGGGC 4184

rabbit  3685                                                                                                      3684
human   4185 CCAGTGCATGGTGGCCAGAGGTGTCACCCAAACCGGCAGGTGCGATTTTGTTAACCCAGCGACGAACTTTCCGAAAAATAAAGACACCTGGTTGCTAACC 4284

rabbit  3685                    3684
human   4285 TGGAAAAAAAAAAAAAAAAA 4304
```

```
human      MGAPACALALCVAVAIVAGASSESLGTEQRVVGRAAEVPGPEPGQQEQLVFGSGDAVELS
mouse      MVVPACVLVFCVA--VVAGATSEPPGPEQRVVRRAAEVPGPEPSQQEQVAFGSGDTVELS
rabbit     MGAPARALALCVAVVVVAGVVWEPAGPERRVVRRAAGVPGLEPSRQEQVVFGSGDTVELS
           * .** .*.:***  :***.  *  * *:*** *** *** **.:***:.*****:****

human      CPPPGGGPMGPTVWVKDGTGLVPSERVLVGPQRLQVLNASHEDSGAYSCRQRLTQRVLCH
mouse      CHPPGGAPTGPTVWAKDGTGLVASHRILVGPQRLQVLNASHEDAGVYSCQHRLTRRVLCH
rabbit     CHLPGGGPLGPTVWVKDGTGLASTDRILVGPQRLQVLNASQEDAGAYSCRQRLTQRVLCH
           *  ***.* *****.******. :.*:*************:**:*.***::***:*****

human      FSVRVTDAPSSGDDEDGEDEAEDTGVDTGAPYWTRPERMDKKLLAVPAANTVRFRCPAAG
mouse      FSVRVTDAPSSGDDEDGEDVAE----DTGAPYWTRPERMDKKLLAVPAANTVRFRCPAAG
rabbit     FIVRLTEAPSSGDDEDGEDEAED----TGAPYWTRPERMDKKLLAVPAANTVRFRCPAAG
           * **:*:************ **     *********************************

human      NPTPSISWLKNGREFRGEHRIGGIKLRHQQWSLVMESVVPSDRGNYTCVVENKFGSIRQT
mouse      NPTPSISWLKNGKEFRGEHRIGGIKLRHQQWSLVMESVVPSDRGNYTCVVENKFGSIRQT
rabbit     NPTPSISWLKNGKEFRGEHRIGGIKLRHQQWSLVMESVVPSDRGNYTCVVENKFGSIRQT
           ************:***********************************************

human      YTLDVLERSPHRPILQAGLPANQTAVLGSDVEFHCKVYSDAQPHIQWLKHVEVNGSKVGP
mouse      YTLDVLERSPHRPILQAGLPANQTAILGSDVEFHCKVYSDAQPHIQWLKHVEVNGSKVGP
rabbit     YTLDVLERSPHRPILQAGLPANQTAVLGSDVEFHCKVYSDAQPHIQWLKHVEVNGSKVGP
           *************************:**********************************

human      DGTPYVTVLKTAGANTTDKELEVLSLHNVTFEDAGEYTCLAGNSIGFSHHSAWLVVLPAE
mouse      DGTPYVTVLKTAGANTTDKELEVLSLHNVTFEDAGEYTCLAGNSIGFSHHSAWLVVLPAE
rabbit     DGTPYVTVLKTAGANTTDKELEVLSLRNVTFEDAGEYTCLAGNSIGFSHHSAWLVVLPAE
           **************************:*********************************

human      EELVEADEAGSVYAGILSYGVGFFLFILVVAAVTLCRLRSPPKKGLGSPTVHKISRFPLK
mouse      EELMETDEAGSVYAGVLSYGVVFFLFILVVAAVILCRLRSPPKKGLGSPTVHKVSRFPLK
rabbit     EELAAAGEAGSVYAGVLSYGAGFFLFILVVGAVTLCRLHSPPKKGLGSPTVHKVSRFPLK
           ***  : ********:****. ********.** ****:**************:******

human      RQVSLESNASMSSNTPLVRIARLSSGEGPTLANVSELELPADPKWELSRARLTLGKPLGE
mouse      RQVSLESNSSMNSNTPLVRIARLSSGEGPVLANVSELELPADPKWELSRTRLTLGKPLGE
rabbit     RQVSLESSSSMNSTTPLVRVARLSSAEGPTLANVSELELPADPQWELSRARLTLGKPLGE
           *******.:**.*.*****:*****.***.*************:*****:**********

human      GCFGQVVMAEAIGIDKDRAAKPVTVAVKMLKDDATDKDLSDLVSEMEMMKMIGKHKNIIN
mouse      GCFGQVVMAEAIGIDKDRTAKPVTVAVKMLKDDATDKDLSDLVSEMEMMKMIGKHKNIIN
rabbit     GCFGQVVMAEAIGIDKDRAAKPVTVAVKMLKDDATDKDLSDLVSEMEMMKMIGKHKNIIN
           ******************:*****************************************

human      LLGACTQGGPLYVLVEYAAKGNLREFLRARRPPGLDYSFDTCKPPEEQLTFKDLVSCAYQ
mouse      LLGACTQGGPLYVLVEYAAKGNLREFLRARRPPGMDYSFDACRLPEEQLTCKDLVSCAYQ
rabbit     LLGACTQGGPLYVLVEFAAKGNLREYLRARRPPGMDYSFDTCRLPEEQLTFKDLVSCAYQ
           ****************:********:********:*****:*: ****** *********

human      VARGMEYLASQKCIHRDLAARNVLVTEDNVMKIADFGLARDVHNLDYYKKTTNGRLPVKW
mouse      VARGMEYLASQKCIHRDLAARNVLVTEDNVMKIADFGLARDVHNLDYYKKTTNGRLPVKW
rabbit     VARGMEYLASQKCIHRDLAARNVLVTEDNVMKIADFGLARDVHNLDYYKKTTNGRLPVKW
           ************************************************************

human      MAPEALFDRVYTHQSDVWSFGVLLWEIFTLGGSPYPGIPVEELFKLLKEGHRMDKPANCT
mouse      MAPEALFDRVYTHQSDVWSFGVLLWEIFTLGGSPYPGIPVEELFKLLKEGHRMDKPASCT
rabbit     MAPEALFDRVYTHQSDVWSFGVLLWEIFTLGGSPYPGIPVEELFKLLREGHRMDKPANCT
           ***********************************************:*********.**

human      HDLYMIMRECWHAAPSQRPTFKQLVEDLDRVLTVTSTDEYLDLSAPFEQYSPGGQDTPSS
mouse      HDLYMIMRECWHAVPSQRPTFKQLVEDLDRILTVTSTDEYLDLSVPFEQYSPGGQDTPSS
rabbit     HDLYMIMRECWHAAPSQRPTFKQLVEDLDRVLTVTSTDEYLDLSAPFEQYSPGGQDSPSA
           *************.****************:*************.***********:**:

human      SSSGDDSVFAHDLLPPAPPSSGGSRT
mouse      SSSGDDSVFTHDLLPPGPPSNGGPRT
rabbit     SSSGDDSVFTHDLLPPAPPSSGGPRT
           *********:******.***.** **
```
